# The Hepatitis Viral Status in Patients With Hepatocellular Carcinoma: a Study of 3843 Patients From Taiwan Liver Cancer Network

**DOI:** 10.1097/MD.0000000000003284

**Published:** 2016-04-18

**Authors:** Il-Chi Chang, Shiu-Feng Huang, Pei-Jer Chen, Chi-Ling Chen, Chao-Long Chen, Cheng-Chung Wu, Cheng-Chung Tsai, Po-Huang Lee, Miin-Fu Chen, Chuan-Mo Lee, Hsien-Chung Yu, Gin-Ho Lo, Chau-Ting Yeh, Chih-Chen Hong, Hock-Liew Eng, John Wang, Hui-Hwa Tseng, Cheng-Hsiang Hsiao, Hong-Dar Isaac Wu, Tseng-Chang Yen, Yun-Fan Liaw

**Affiliations:** From the Liver Research Unit, Chang Gung Memorial Hospital Linko Branch, Chang-Gung University, Taoyuan, Taiwan (I-CC, C-CH, Y-FL), Institute of Molecular and Genomic Medicine, National Health Research Institutes, Miaoli, Taiwan (I-CC, S-FH, C-CH), Department of Pathology, Chang Gung Memorial Hospital Linko Branch, Taoyuan, Taiwan (S-FH), Graduate Institute of Clinical Medicine, National Taiwan University College of Medicine, Taipei, Taiwan (P-JC, C-LC), Department of General Surgery, Chang Gung Memorial Hospital Kaohsiung Branch, Chang-Gung University, Kaohsiung, Taiwan (C-LC), Department of General Surgery, Taichung Veteran General Hospital, Taichung, Taiwan (C-CW), Department of General Surgery, Kaohsiung Veteran General Hospital, Kaohsiung, Taiwan (C-CT), Department of General Surgery, National Taiwan University Hospital, Taipei, Taiwan (P-HL), Department of General Surgery, Chang Gung Memorial Hospital Linko Branch, Chang-Gung University, Taoyuan, Taiwan (M-FC), Department of Hepato-gastroenterology, Chang Gung Memorial Hospital Kaohsiung Branch, Chang-Gung University, Kaohsiung, Taiwan (C-ML), Department of Hepato-gastroenterology, Kaohsiung Veteran General Hospital, Kaohsiung, Taiwan (H-CY, G-HL), Department of Hepato-gastroenterology, Chang Gung Memorial Hospital Linko Branch, Chang-Gung University, Taoyuan, Taiwan (C-TY), Department of Pathology, Chang Gung Memorial Hospital Kaohsiung Branch, Chang-Gung University, Kaohsiung, Taiwan (H-LE), Department of Pathology, Taichung Veteran General Hospital, Taichung, Taiwan (JW), Department of Pathology, Kaohsiung Veteran General Hospital, Kaohsiung, Taiwan (H-HT), Department of Pathology, National Taiwan University Hospital, Taipei, Taiwan (C-HH), Department of Applied Mathematics and Institute of Statistics, National Chung-Hsing University, TaiChung, Taiwan (H-DIW, T-CY).

## Abstract

Hepatocellular carcinoma (HCC) is the leading cancer death in Taiwan. Chronic viral hepatitis infections have long been considered as the most important risk factors for HCC in Taiwan. The previously published reports were either carried out by individual investigators with small patient numbers or by large endemic studies with limited viral marker data. Through collaboration with 5 medical centers across Taiwan, Taiwan liver cancer network (TLCN) was established in 2005. All participating centers followed a standard protocol to recruit liver cancer patients along with their biosamples and clinical data. In addition, detailed viral marker analysis for hepatitis B virus (HBV) and hepatitis C virus (HCV) were also performed. This study included 3843 HCC patients with available blood samples in TLCN (recruited from November 2005 to April 2011). There were 2153 (56.02%) patients associated with HBV (HBV group); 969 (25.21%) with HCV (HCV group); 310 (8.07%) with both HBV and HCV (HBV+HCV group); and 411 (10.69%) were negative for both HBV and HCV (non-B non-C group). Two hundred two of the 2463 HBV patients (8.20%) were HBsAg(-), but HBV DNA (+). The age, gender, cirrhosis, viral titers, and viral genotypes were all significantly different between the above 4 groups of patients. The median age of the HBV group was the youngest, and the cirrhotic rate was lowest in the non-B non-C group (only 25%). This is the largest detailed viral hepatitis marker study for HCC patients in the English literatures. Our study provided novel data on the interaction of HBV and HCV in the HCC patients and also confirmed that the HCC database of TLCN is highly representative for Taiwan and an important resource for HCC research.

## INTRODUCTION

Liver cancer is the fifth most common cancer in men and the seventh in women worldwide, and it is also the second most frequent cause of cancer death in the world.^1,2^ Hepatocellular carcinoma (HCC) accounts for 75∼85% of all primary liver cancers.^1^ In addition, there is great geographical variations in the incidence of HCC, with 75 to 80% HCC patients occurred in Africa and Asia, where are also the endemic areas of chronic viral hepatitis.^1–4^ The major risk factors of HCC include chronic viral hepatitis, cirrhosis, alcoholism, and fatty liver diseases.^1–5^ In Taiwan, the vaccination program against hepatitis B virus (HBV) infection was initiated since 1984 and has reduced the incidence of HCC in children and adolescent successfully,^6,7^ but HCC still ranks the first in cancer incidence and mortality in men in Taiwan, with more than 7000 individuals died of HCC annually.^8^ Although the major viral and environmental risk factors have been identified, the oncogenic pathways leading to malignant transformation of liver cells remains unclear. In addition, as most of the HCC patients were diagnosed at an advanced stage or with advanced cirrhosis, the prognosis of HCC patients remained poor, with the median 5-year survival rate less than 50%.^2^ Investigators from different institutes in Taiwan already have performed researches in HCC extensively and have demonstrated that HBV and hepatitis C virus (HCV) are the major etiologic agents for HCC.^9–13^ The above individual projects have produced some very interesting results that require more comprehensive study to confirm or to advance. However, as most previous studies were carried out by individual institute, the study cases and samples are limited and, sometimes, not so representative. In addition, the recent advances in genomics and proteomics led to discoveries of numerous promising new biomarkers in cancer research. The availability of large number, high-quality HCC tissues to validate those biomarkers has become a major bottleneck for HCC researchers in Taiwan.

To address these problems, we need a large number of patients from different parts of Taiwan for detailed analyses. Therefore, Taiwan Liver Cancer Network (TLCN) was established to coordinate the major medical centers in Taiwan to collect tumor tissues and blood samples of liver tumor patients along with comprehensive clinical and epidemiological data.

In this report, we would like to present a detailed viral marker analysis, which included HBsAg, HBV DNA, HBV genotype, anti-HCV, HCV RNA, and HCV genotypes along with their clinic-pathological features on a total of 3843 HCC patients in TLCN. These patients were recruited from November 1, 2005, to April 30, 2011. Even though several large-scaled HCC patient studies in Taiwan have been published before,^14–17^ none of them have so detailed viral marker data as in this report. These information will help investigators to appreciate that TLCN has a most important resource for liver cancer research in Taiwan.

## METHODS

### Establishment and Operation of TLCN

**FIGURE 1:**
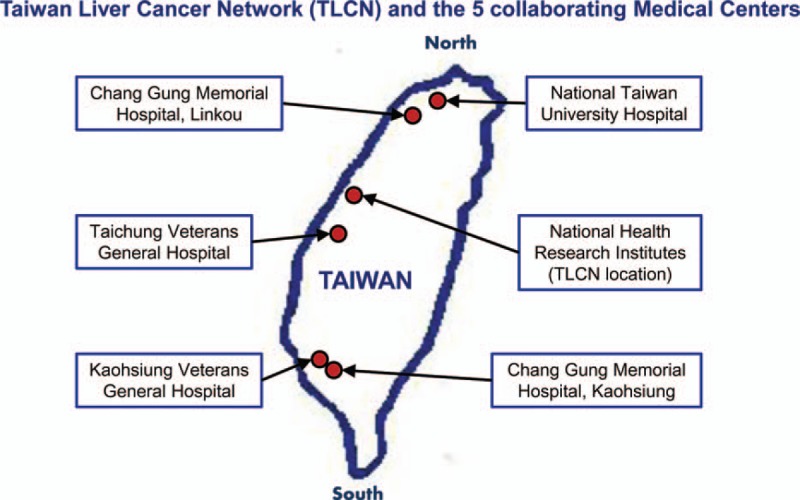
The location of the 5 collaborating medical centers and the central tissue bank at National Health Research Institutes are shown on the map of Taiwan.

To recruit liver cancer patients with various socioeconomic, ethnical, and lifestyle backgrounds as well as regional representativeness, TLCN was established in a central facility at National Health Research Institutes (NHRI) in collaboration with 5 medical centers, which are located in the northern (National Taiwan University Hospital and Chang Gung Memorial Hospital Linko Branch), central (Taichung Veteran General Hospital), and southern parts (Chang Gung Memorial Hospital Kaohsiung Branch and Kaohsiung Veteran General Hospital) of Taiwan, respectively (Figure 1). A team consists of hepatologist, surgeon, and pathologist was formed in each collaborating medical center to collect biosamples and clinical information. A well-trained research nurse was stationed at each collaborating hospital to help with all the collection procedures. All participating centers follow a common protocol to collect biosamples, which include blood, fresh frozen tissue, and paraffin tissue blocks, as well as the patients’ clinical, pathological, and epidemiological information. Standardized questionnaire and clinical abstract were designed prospectively and used for all patients recruited in TLCN.

For collection of biosamples, all of the liver tumor and paired nontumor tissue were snap frozen immediately after resection and stored in a −80°C deep freezer at each collaborating hospital. The blood samples were separated into serum, plasma, buffet coat immediately, and stored in the −80°C deep freezer rapidly, too. All biosamples obtained from each hospital were kept frozen and shipped with dry ice to the central tissue bank at NHRI per month. Then, the serum samples were sent to the core viral laboratory at the liver Unit of Chang-Gung Memorial Hospital for HBV DNA and HCV RNA assays periodically. Serum hepatitis B surface antigen (HBsAg) and anti-HCV antibody would also be tested, if the data were not found in the medical records. The clinical abstract from the medical records and questionnaires from the patients were sent to the central database office of TLCN at NHRI regularly and handled by independent technicians and stored in a separated place away from the tissue bank. The TLCN office was in charge of organization and warehousing of the patients’ database. The follow-up data and survival status of the patients were regularly updated according to the medical records and the death record of Department of Health of Taiwan. A Working Committee consisted of 10 experts in liver research as well as biomedical research was formed. All except 3 of the members did not belong to any of the 5 medical centers. The mission of the Working Committee was to assess and decide issues related to the usage of the biosamples and database of TLCN and to review all applications for TLCN biosamples or database. This study protocol has been approved by the Institutional Review Board of all 5 Medical Centers, and later approved by Minister of Health and Welfare of Taiwan in year 2014. Signed informed consents were obtained before the collection of the specimens and data in all patients.

### Patients

From November 1, 2005, to April 30, 2011, a total of 5170 patient samples (4941 patients) were collected in TLCN. Among them, 4067 patients (82.31%) were diagnosed as HCC, and 3843 patients have available serum sample for a complete viral marker analysis. These patients were included as the study subjects in this analysis.

### Detection of Viral Makers

Test results of HBsAg and anti-HCV of the patients were obtained from the medical records in the majority of patients. All were assayed in the central clinical laboratories of the respective medical centers. For patients without these data, and had sufficient stored serum samples, HBsAg, and anti-HCV were assayed retrospectively by the central clinical laboratory of Chang-Gung Memorial Hospital Linko branch. Elecsys HBsAg II test kit and Elecsys Anti-HCV assay kit (Roche Applied Science, Hannheim, Germany) were used, respectively.

As patients with negative HBsAg might still be positive for HBV DNA, quantification of HBV DNA was performed on all patients who had blood samples. Serum HBV DNA was measured using the Cobas Amplicor HBV monitor test kit (Roche Applied Science, Hannheim, Germany). The detection limits was 315 copies/mL before year 2011, and thereafter, HBV AmpliPrep/COBAS TaqMan HBV test kit was used (Roche Applied Science, Hannheim, Germany). The detection limit was 12 IU/mL or 69 copies/mL.

The HCV RNA level was assayed only for patients who were positive for anti-HCV. HCV RNA was measured using AmpliPrep/COBAS TaqMan HCV test kit (Roche Applied Science, Hannheim, Germany). The detection limit was 15 IU/mL or 41 copies/mL.

HBV genotype was determined using SMITEST HBV Genotyping Kit (Medical & Biological Laboratories Co., Ltd, Boston, MA), which uses a combination of PCR and ELISA methods. Specimen preparation and process were according to the manufacturer's instructions. For HCV genotyping, the INNO-LiPA (line probe assay) method was used. Specimen preparation and process were according to the manufacturer's instructions.

### Statistical Analysis

To examine the differences in the major clinicopathological variables, frequencies and proportions are compared by conventional Chi-square association test. For multiple groups of age data, it was done by Kruskal-Wallis test and Kolmogorov–Smirnov test. A 2-sided *P* value less than 0.05 was considered as statistically significant.

## RESULTS

All HCC patients were classified as HBV, HCV, or HBV+HCV group according to seropositivity of HBsAg and/or anti-HCV. For patients negative for both seromarkers, those with detectable serum HBV DNA were also included as HBV group, while patients with undetectable HBV DNA were classified as non-B non-C group. Patients who had no HBsAg and anti-HCV data in the medical records and had no blood sample for further viral study were excluded from this analysis.

### Chronic HBV and HCV Infections Accounting for Major Etiology for HCC but Differing Among the 5 Hospitals

Among the 4067 HCC patients recruited in TLCN from November 1, 2005, to April 30, 2011, there were 3843 patients with available blood sample for complete viral marker studies. The HBV infection accounts for 56.02%; HCV for 25.21%; dual infection for 8.07%; and non-B/non-C for 10.69%. Although the numbers of patient with HBV were higher than HCV in all of the 5 medical centers, the proportion of HBV and HCV was significantly different among the 5 medical centers (*P* = 0.0000) (Table 1). Patients recruited from Taichung Veteran General Hospital (in the central part of Taiwan) had the highest proportion of HCV patients (35.57%), while the National Taiwan University Hospital (in the north part of Taiwan) had the lowest proportion of HCV patients (17.52%). When we compared the viral genotype patterns, the HBV genotypes were also significantly different for the patients with pure HBV infection (*P* = 0.0000), even though the numbers of genotype B patients were all higher than genotype C in the 5 medical centers (Table 2). In contrast, the HCV genotype patterns in the 5 medical centers had no significant difference (*P* = 0.1120, detailed data not shown).

**TABLE 1:**
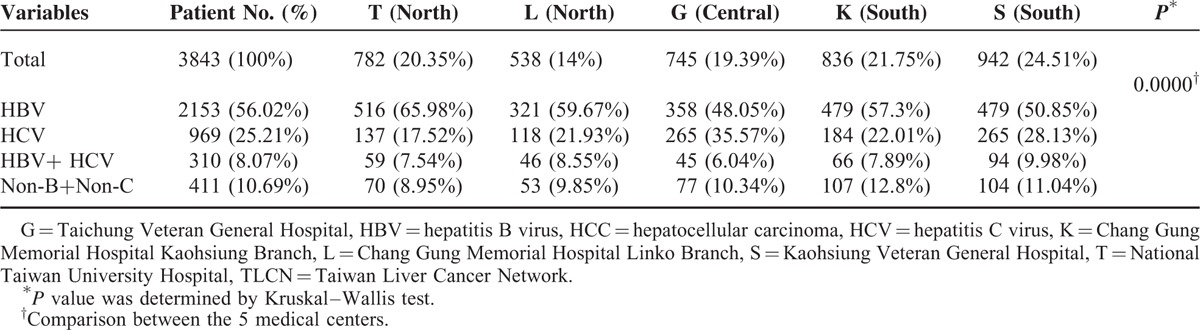
The Proportion of HBV and HCV Infections of the 3843 HCC Patients Among the 5 Medical Centers in TLCN

**TABLE 2:**
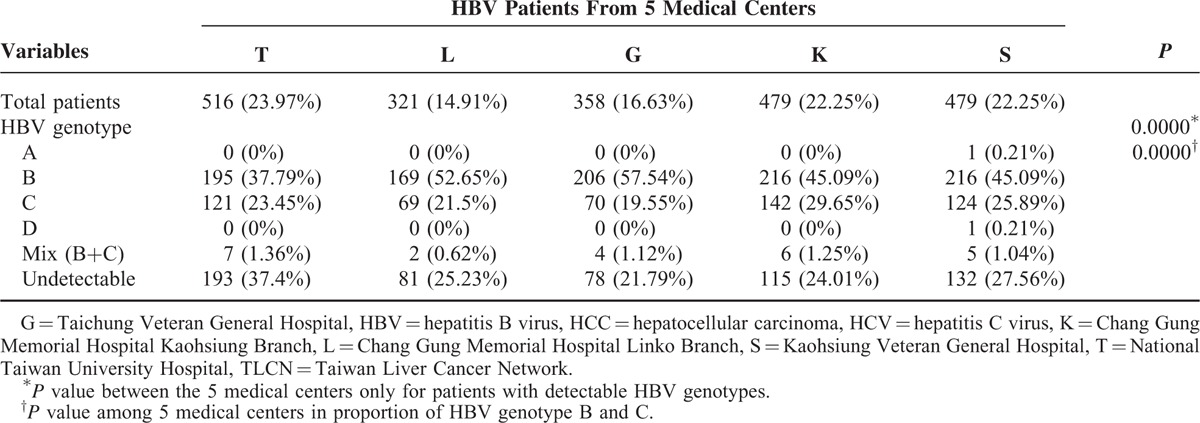
HBV Genotype Distribution of 2153 HBV Patients Among the 5 Medical Centers in TLCN

### The Detailed Viral Data Analysis Among 3843 HCC Patients

There were 2153 patients (56.02%) with pure HBV infection and classified as HBV group; 969 patients (25.21%) as HCV group; 310 patients (8.07%) as HBV+HCV group; and 411 patients (10.69%) were non-B non-C group (Table 3). For HBV DNA level, 40.08% of the HBV group still had high HBV DNA levels (defined as **≥**2 × 10^**4**^ IU/mL). This cut-off value was according to the American Association for the Study of Liver Diseases (AASLD) guidelines for chronic hepatitis B in 2007.^18^ In that guidelines, HBV DNA levels **≥**2 × 10^4^ IU/mL was a diagnostic criterion for active chronic hepatitis B.^18^ For HCV group, 66.15% of patients still had high HCV RNA levels (defined as ≥2.3 × 10^4^ IU/mL). This cut-off value was according to the study by Lee et al.^19^ They found that 2.3 × 10^4^ IU/mL was the best cut-off for predicting future HCC development in a cohort of 972 patients with chronic HCV infection.^19^ Genotype B and genotype I was the major genotype for HBV and HCV patients, respectively. Only 164 of the 3843 HCC patients have received antiviral treatment before the operation. The 164 patients included 138 (6.41%) of the 2153 HBV patients, 11 (1.14%) of the 969 HCV patients, and 15 (4.84%) of the 310 HBV+HCV patients, respectively. Among the 164 patients, 20 (14.49%) of HBV group patients still have HBV DNA level ≥2 × 10^4^ IU/mL, and 6 (54.55%) of HCV group patients still had high HCV RNA level (≥2.3 × 10^4^ IU/mL). For HBV+HCV patients, only 2 (13.33%) and 2 (13.33%) patients still had high HBV DNA and HCV RNA levels, respectively. The HBV DNA was undetectable in 31 (22.46%) of the 138 HBV patients.

**TABLE 3:**
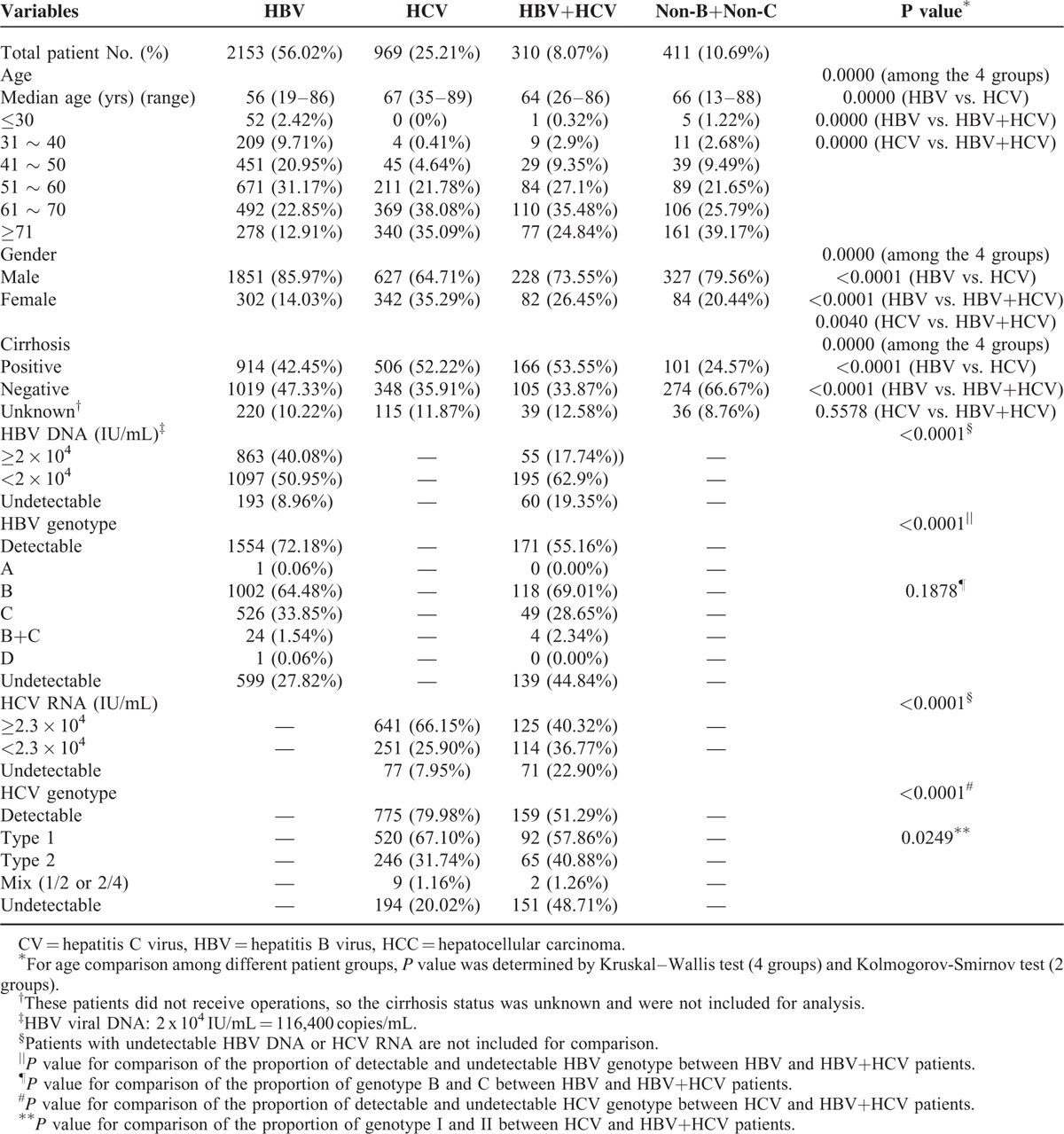
The clinical characteristics and the status of HBV and HCV infection in the 3843 HCC patients

### The Clinical Characteristics of the 4 Patient Groups

The clinical characteristic features of the 3843 HCC patients are summarized in Table 3. The HBV group were much younger than the other 3 (mean age 56 vs 64–67 year-old), and more likely male (86%). The proportion with cirrhosis was similar (43–53%) among the 3 virus-related HCCs, but was much lower in non-B non-C HCCs (only 25%). It appears that the clinical characteristics of the patients in the HBV+HCV group were in between the HBV and HCV groups, such as age, gender, and cirrhotic rate. The HBV DNA or HCV RNA levels were more frequently undetectable in HBV+HCV group than HBV group (19.35% vs 8.96%, *P* < 0.0001) or HCV group (22.90% vs 7.95%, *P* < 0.0001).

### HBV DNA and HCV RNA Levels in the 310 HBV+HCV Associated HCC Patients

Comparison of the HBV DNA and HCV RNA levels in the 310 HBV+HCV associated HCC patients is summarized in Table 4. For patients with HBV DNA level ≥2 × 10^4^ IU/mL, only 8 patients (8/55, 14.55%) also had high HCV RNA levels (≥2.3 × 10^4^ IU/mL). In contrast, when HBV DNA level was undetectable, up to 33 patients (33/60, 55%) had high HCV RNA levels (*P* = 0.0001).

**TABLE 4:**
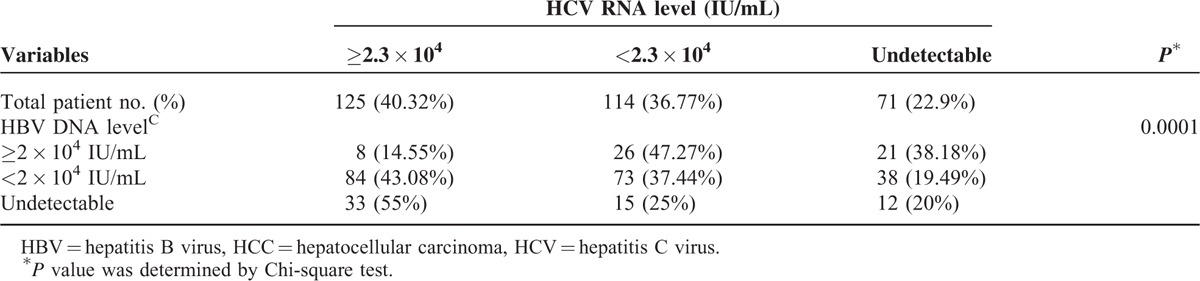
The HBV DNA and HCV RNA Levels of the 310 HCC Patients With Both HBV and HCV Infections

### HBV HCC Patients With Negative HBsAg and Detectable HBV DNA

We also identified 202 patients who were negative for HBsAg, but had detectable serum HBV DNA. Among them, 115 patients had HBV mono-infection and 87 had both HBV and HCV. The clinical characteristics are summarized in Table 5. It is worthy of attention that 22 of the 202 patients still had an HBV DNA level ≥2 × 10^4^ IU/mL.

**TABLE 5:**
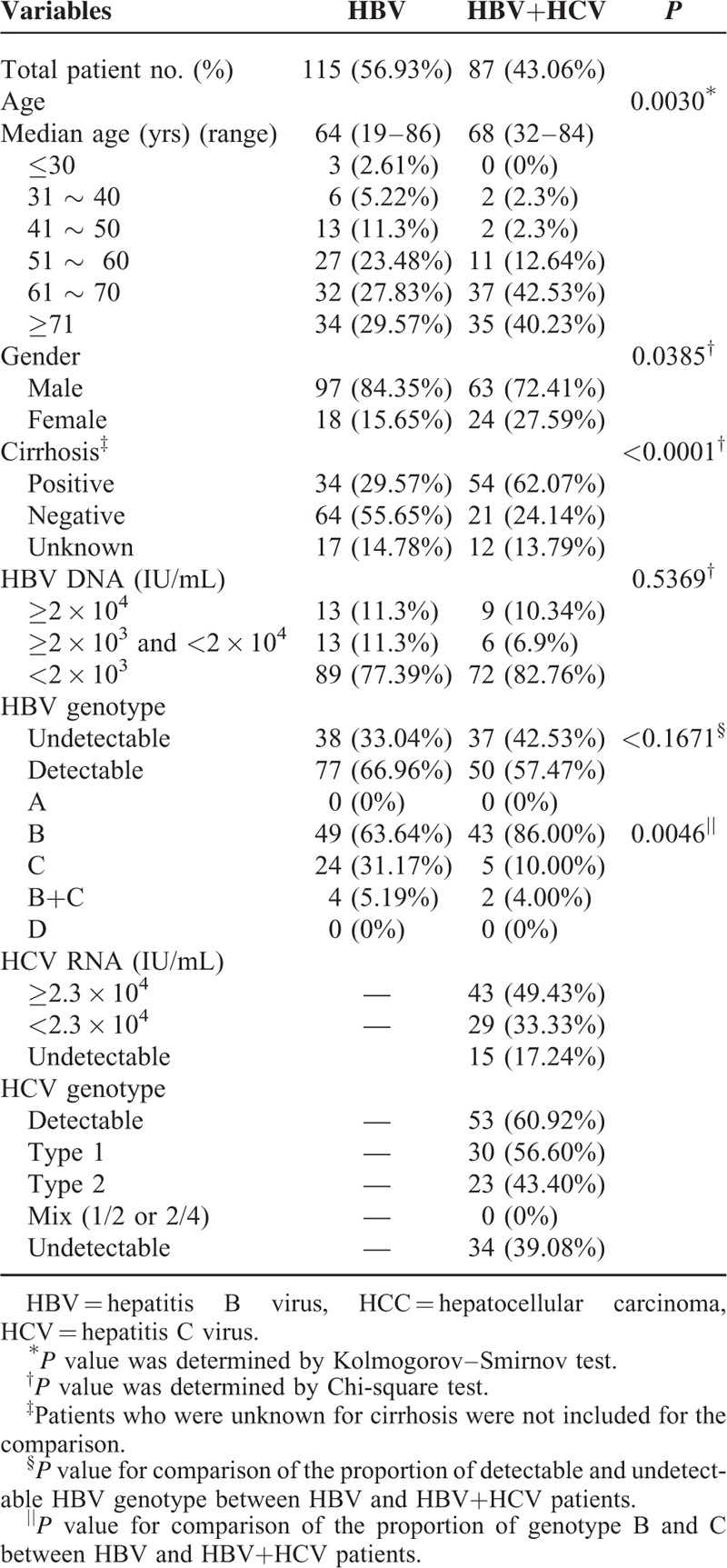
The Clinical Characteristics of 202 HCC Patients who were HBsAg (-) but HBV DNA (+)

### HCC Patients With Positive anti-HCV Antibody but Undetectable HCV RNA

Among the 1215 patients positive for anti-HCV antibody, the HCV RNA was undetectable in 143 patients. Their clinicopathological characteristics were compared with patients who had detectable HCV RNA and summarized in Table 6. The age, gender, and cirrhotic rate were all significantly different. Patients with undetectable RNA were younger, more male, with lower cirrhotic rate, and had more HBV coinfection.

**TABLE 6:**
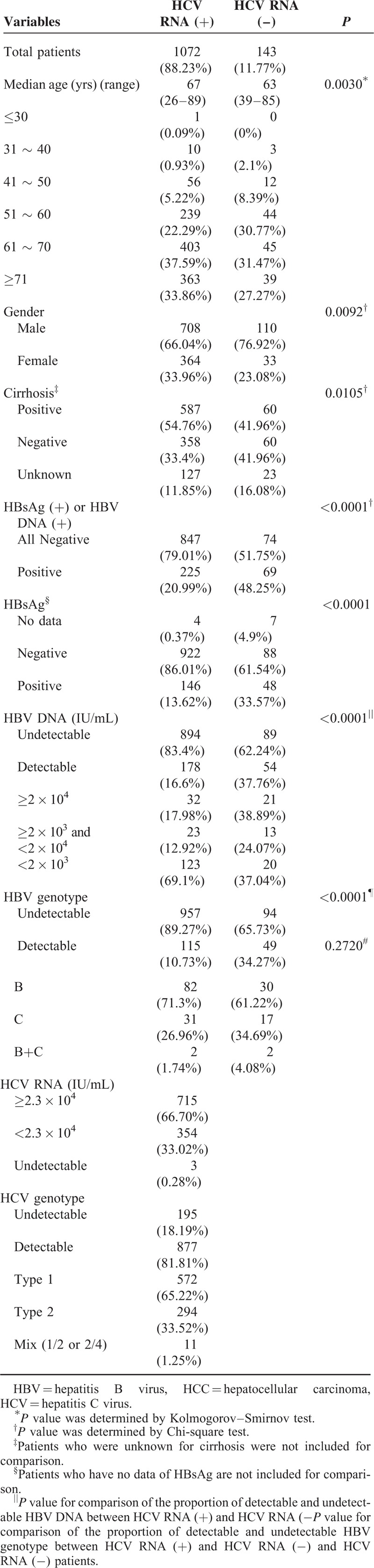
Clinical Characteristics of 1215 HCC Patients who were Positive for anti-HCV Antibody

### HCC Patients Negative for Both HBV and HCV (Non-B Non-C group)

There were 411 (10.69%) patients who were negative for all viral markers. To search the risk factors associated with hepato-carcinogenesis, we have collected the data, including body mass index (BMI), hypertension, fatty liver (by sonography), diabetes, smoking and drinking history, serum auto-antibodies, cholesterol and triglyceride levels, etc., of these 411 patients. However, majority of these patients had no fatty change (314, 76.4%) by sonography examination and were negative or normal for all the above risk factors.

## DISCUSSION

This is the largest detailed viral status study in HCC patients in the English literatures. It confirms that HBV (56.02%) and HCV (25.21%) prevailed in Taiwan HCC patients. It also revealed that dual infection (8.07%) played a significant role, a unique feature in Taiwan. Only 10.69% HCC patients had no evidence of HBV or HCV infection. As majority of the HCC patients in TLCN were operable patients and with better functional class, the cirrhotic rate in these patients would be lower than the whole HCC populations in Taiwan.

For the age distribution, the median age of HBV-HCC patients was youngest among the 4 groups, which is consistent with previous reports from Taiwan^10,14,17^ and Japan.^20^ Lu et al^14^ proposed that the age difference in patients between HBV-HCC and HCV-HCC was due to early HBV infection in perinatal period, while HCV infection is acquired in adulthood. The median age of HBV+HCV group was between HBV-HCC patients and HCV-HCC patients (Table 3). Similar observation was also found in 2 previously studies.^14,17^ It was considered to be from HBV infection in early time of life and superimposed HCV infection in latter time of life.^14^ In addition, the age distribution of HBV group and HCV group was also quite different. In HBV group, the case number was decreased after 60 years of age. This trend was similar to the result of Chen et al.^21^ In contrast, the case number in the HCV group increased with age, being highest after 60 years of age. This trend has also been reported before.^21,22^ Of interest is the non-B non-C group (411 patients). Their median age was quite old, almost similar to HCV group (66 vs. 67), but had the lowest cirrhotic rate (24.57%). The gender distribution is also quite different among the 4 groups. The percentage of female patients is lowest in the HBV group (14.03%) and highest in the HCV groups (35.29%), which is also similar to previous reports.^14,17^ The female patients in the non-B non-C groups were also quite low (20.44%), suggesting that the female sex hormone may still play a protective role even without viral infection. As we have tested both HBsAg and HBV DNA in all patients, the non-B non-C group of patients in this study should be really virus-free. However, majority of these patients had no fatty change (314, 76.4%) and were negative or normal for the common metabolic risk factors, such as BMI, hypertension, diabetes, smoking and drinking history, serum auto-antibodies, cholesterol and triglyceride levels, etc. Further comparison with age and gender-matched control from the HBV and HCV groups of HCC patients for the above risk factors is ongoing.

In patients with chronic viral hepatitis, if they have both HBV and HCV infections, the 2 viruses may interfere with each other.^23–27^ In the present study, the viral titers of the 2 viruses among the 310 HBV+HCV HCC patients were quite similar to the situation in the chronic hepatitis patients. The higher the HBV viral titers, the lower of the HCV RNA, and vice versa.

HBsAg seroclearance is usually considered as clearance of HBV infection.^28,29^ In previously published reports with very large number of HCC patients,^14,17^ the non-B non-C patients were defined by HBsAg (-) and anti-HCV antibody (-) only, which could include a significant number of occult HBV patients, as the current study has identified 202 patients (8.20%) who were HBsAg(-), but HBV DNA (+) among the 2463 HBV patients. In addition, 22 of the 202 patients still had high HBV DNA level. High serum HBV DNA accompanied with HBSAg seroclearance has been reported previously.^30^ These patients might have HBS gene mutations, which resulted in detection failure of the HBsAg.^31,32^

The limitations of this study are that we mainly collected HCC patients who were operable. Thus, these 3843 patients would have better functional class, lower cirrhotic rate, and more in early tumor stages than the general HCC populations. As not all HCC patients in each hospital were recruited into TLCN (some patients refused or refused to donate their blood samples), so there might be some sampling bias. However, as the patient number from each hospital was all quite large and the 5 hospitals were across Taiwan, we are confident that this detailed viral marker study of 3843 HCC patients is not only highly representative for Taiwan but also provided novel data on the interaction of HBV and HCV in the HCC patients. By year 2015, TLCN has successfully recruited 7600 liver tumor patients after it was established for 10 years. It has become an important resource for future HCC research.
